# Editorial: Genetics of Apicomplexans and Apicomplexan-Related Parasitic Diseases

**DOI:** 10.3389/fgene.2022.875778

**Published:** 2022-03-10

**Authors:** Moses Okpeku, Matthew A. Adeleke, Jun-Hu Chen

**Affiliations:** ^1^ Discipline of Genetics, School of Life Sciences, University of Kwazulu-Natal, Durban, South Africa; ^2^ National Institute of Parasitic Diseases, Chinese Center for Diseases Control and Prevention (Chinese Center for Tropical Diseases Research), Shanghai, China; ^3^ National Health Commission of the People’s Republic of China (NHC) Key Laboratory of Parasite and Vector Biology, Shanghai, China; ^4^ World Health Organization (WHO) Collaborating Centre for Tropical Diseases, Shanghai, China; ^5^ National Center for International Research on Tropical Diseases, Shanghai, China

**Keywords:** apicomplexan-related parasitic diseases, genetics, malaria, theileriosis, tick, anopheles

Apicomplexa are a large phylum of parasitic organisms. They are mostly unicellular and spore-forming in nature. Majority are parasites of humans, livestock, wild and domesticated animals, birds, and aquatic organisms; responsible for some parasitic diseases of serious economic importance like babesiosiosis, cryptosporidiosis, cystoisosporiasis, malaria and toxoplasmosis. Although great efforts in epidemiology, vector control, and use of medicinal drugs have been very helpful in reducing disease burden of Apicomplexa-based disease: control, prevention, and elimination of these diseases is dependent on a better understanding of their genetics, mechanisms of infection, and immunity control methods (such as drugs/vaccines and vector control methods). Furthermore, knowledge of host-vector-parasite interactions is vital for stimulating new concepts and tools for control, management, and policy formulation. This issue puts together some current researches involving apicomplexan.

The phylum Apicomplexa is entirely composed of obligate intracellular parasites, causing a plethora of severe diseases in humans, wild and domestic animals. Mitochondria are vital organelles of eukaryotic cells, participating in key metabolic pathways. The mitochondria in Apicomplexa is as a promising source of undiscovered drug targets, and it has been validated as the target of atovaquone, a drug currently used in the clinic to counter malaria, but highly diverse (Berná et al.). In this issue, Berná et al. extensively reviewed mitochondrial genomic and mechanistic features found in evolutionarily related alveolates, as well as common and distinct origins of the apicomplexan mitochondria peculiarities, with a focus on genomic insight gained from second and third generation sequencing technologies.

Malaria accounts to be responsible for large morbidity and mortality, particularly in sub-Saharan Africa where the greatest burden of the disease is borne. Chloroquine (CQ) and sulfadoxine–pyrimethamine (SP) are core drugs used in malaria control. However, a core challenge is the emergence and spread of drug resistance. Roux et al. traced the spread of CQ and SP resistance in sub-Saharan Africa, catalogued different control strategies adopted to control the spread of resistant parasites. This review suggests that close monitoring is required to prevent establishment and spread of resistance and to detection strategies for tracking possible reintroduction of drug resistance to specific anti-malarial drugs, especially as the region works towards malaria elimination. In another contribution, Yang et al. focused on *Plasmodium falciparum* surface-related antigen (SRA) located on the surfaces of gametocyte and merozoite. The SRA possess structural and functional characteristics that make them potential targets for multistage vaccine development. However, little information is available regarding the genetic polymorphism of *pfsra*. The study determined genetic variation in *pfsra* sequences generated from 74 samples collected from migrant workers who returned to China from 12 malaria endemic countries in Africa. Average pairwise nucleotide diversities (π), haplotype diversity (*Hd*), average number of nucleotide differences (*k*) of *P. falciparum sra* gene were 0.00132, 0.770, and 3.049, respectively. The ratio of non-synonymous (*dN*) to synonymous (*dS*) substitutions across sites (*dN*/*dS*) was 1.365. Neutrality tests showed polymorphism of *pfsra* genes maintained by positive diversifying selection, implicating this gene as a potential target of protective immune responses and a vaccine candidate (Yang et al.). In a separate study from Pakistan, Khan et al. investigated the molecular diversity of *P. falciparum* based on msp-1 and msp-2 genes. The study concluded that the *P. falciparum* populations and allelic variants of msp-1 and msp-2 in the study region are highly polymorphic and diverse. HSP101 is related to host cell remodelling of malaria parasites. Kreutzfeld et al. generated transgenic *P. berghei* parasite lines that restore liver stage expression of HSP101, which were able to complete the life cycle, but failed to export PEXEL-proteins in early liver stages. The results suggest that PTEX-dependent early liver stage export cannot be restored by addition of HSP101, indicative of alternative export complexes or other functions of the PTEX core complex during liver infection.

*Theileria parva* is a protozoan parasite transmitted by the brown-eared ticks, *Rhipicephalus appendiculatus* and *R. zambeziensis*. The parasite has two transmission modes; cattle–cattle and buffalo–cattle transmission. Maboko et al., using Illumina HiSeq whole genome sequence data, compared genetic diversity in *T. parva* sampled from East and Southern Africa. Buffalo-derived strains had higher genetic diversity, with twice the number of variants compared to cattle-derived strains, with possible geographic sub-structuring. They conclude that *T. parva* strains circulating in South Africa are buffalo derived, and associated with corridor disease, but further work is needful for identifying host specificity (Maboko et al.), but suggested that East Coast fever (ECF) was not circulating in South African buffaloes. In a separate study, Allan et al., compared the complex interplay between *T. parva* populations in buffalo and cattle, revealing the extent of the significant genetic diversity in the buffalo *T. parva* population, the limited sharing of parasite genotypes between the host species, and highlighting that a subpopulation of *T. parva* is maintained by transmission within cattle. The results emphasize the importance of obtaining a fuller understanding of buffalo *T. parva* population dynamics in particular, for holistic understanding of *T. parva* genetics to enable a realistic assessment of the extent of buffalo-derived infection risk in cattle. *T. annulata* which causes tropical theileriosis, is a major impediment to improving cattle production in Sudan (Salih et al.). Salih et al., investigated the genetic structure of four *T. annulata* populations in Sudan revealed substantial intermixing, with only two groups exhibiting regional origin independence. The results show that the live schizont attenuated vaccine, Atbara strain may be acceptable for use in all Sudanese regions where tropical theileriosis occurs.

The distribution, genetic diversity, and zoonotic potential of *Cryptosporidium*, *Enterocytozoon bieneusi*, and *Giardia duodenalis* in wildlife are poorly understood. Zhang et al. reported the first molecular epidemiological investigation of three pathogens in wildlife from Zhejiang province and Shanghai city, China. Sequence analyses revealed five known (BEB6, D, MJ13, SC02, and type IV) and two novel (designated SH_ch1 and SH_deer1) genotypes of *E. bieneusi*. The overall results suggest that wildlife act as host reservoirs carrying zoonotic *E. bieneusi* and *G. duodenalis*, potentially enabling transmission from wildlife to humans and other animals (Zhang et al.). Cabarcas et al. performed a thorough comparison of 100+ *de novo* assembled genomes of *C. hominis*. After quality genome filtering, a comprehensive phylogenomic analysis allowed them to discover that *C. hominis* encompasses two lineages with continental segregation. These lineages were named as *C. hominis* Euro-American (EA) and the *C. hominis* Afro-Asian (AA) lineages based on the observed continental distribution bias.

Ticks are ectoparasites of humans and animals, and important vectors of apicomplenxan parasites. In this issue, Cao et al. identified 189 microbial genera and 284 species from different tick populations, using mNGS Illumina HiSeq platform, identified at taxa level (*Anaplasma*, *Rickettsia*, *Ehrlichia* species), genus (*Wolbachia*), as well as species (*Candidatus* Entotheonella) levels. The results of this study provide insights into possible profiling of known and novel tick-borne pathogens in the study region. Metagenomics next-generation sequencing (mNGS) has been suggested to be useful for rapid and accurate characterization of microorganism abundance and diversity. The promotion of NGS usage in apicomplexan research holds promises for advancement in apicomplexan profiling and characterization of known and novel types. Understanding genetic architecture of such is key in combating diseases transmitted by these myriads of microbes that co-habit the planet with us. *Anopheles coluzzii* is a major African malaria vector in the Gambiae species complex. Holm et al. used a massively parallel reporter assay, Self-Transcribing Active Regulatory Region sequencing (STARR-seq), to generate the first comprehensive genome-wide map of transcriptional enhancers in *A. coluzzii* and reported a catalog of 3,288 active genomic enhancers. This enhancer catalogue will be valuable in identifying regulatory elements that could be employed in vector manipulation.

Apicomplexan-related parasitic diseases are ancient diseases, great deal of effort invested into control and prevention of the diseases from the globe has seen measurable success, but the diseases persist. The unique multifaceted nature of the apicomplexan parasites, the potential to infect and cross infect more than one host has made viable vaccine intervention a difficult task. The deployment of genomic techniques is yielding useful information that can be explore for predicted allele-based vaccines, concocted into multi-target vaccine coupled with potent drugs is promising.

